# A self-control training app to increase self-control and reduce aggression – A full factorial design

**DOI:** 10.1016/j.invent.2021.100392

**Published:** 2021-04-20

**Authors:** Hanneke Kip, Marcia C. Da Silva, Yvonne H.A. Bouman, Lisette J.E.W.C. van Gemert-Pijnen, Saskia M. Kelders

**Affiliations:** a Centre for eHealth and Wellbeing Research, Department of Psychology, Health & Technology, University of Twente, Enschede, the Netherlands; b Department of Research, Stichting Transfore, Deventer, the Netherlands; c Faculty of Medical Sciences, University of Groningen, University Medical Center Groningen, Groningen, the Netherlands; d Optentia Research Focus Area, North-West University, Vanderbijlpark, South Africa

**Keywords:** Self-control training, Aggression, Mobile app, eHealth

## Abstract

**Background:**

Research has shown that self-control training (SCT) is an effective intervention to increase self-control and behaviour driven by self-control, such as reactive aggression. We developed an app that offers SCT by asking users to use their non-dominant hand for daily tasks, and aimed to examine whether participants that received SCT via app or e-mail, and received either one daily task or five tasks at once, improved more in self-control and decreased in aggression compared to each other and a control group.

**Methods:**

The design of this study was based on a pilot study in which a first version of the SCT app was developed and tested with students via a pretest-posttest design. Based on the outcomes of the pilot study, a 2 × 2 full factorial design (*N* = 204) with control group (*n* = 69) was used, with delivery via e-mail versus app and receiving one daily task versus five at once as factors. During four measuring points, self-control was assessed via the Brief Self-Control Scale (BSCS) and the Go/No-Go task, aggression was assessed using the Brief Aggression Questionnaire (BAQ). In the final questionnaire, open-ended questions were asked to gain insight into the app's points of improvement. Quantitative data were analysed using repeated measures linear mixed models, qualitative data were analysed via inductive coding.

**Results:**

While no interaction effects were found, analyses showed that only the BSCS-scores of participants that used the app significantly improved over time (F[3, 196.315] = 4.090, *p* = .008), no improvements were observed in the e-mail and control condition. No meaningful differences in aggression, the Go/No-Go task, and between the one- and five-task conditions and control groups were found. Qualitative data showed that while the opinions on SCT-tasks differed, participants were overall satisfied with the intervention, but wanted more reminders.

**Conclusions:**

The results of this study showed that an SCT app has the potential to bolster self-control. No convincing effects on aggression were found in this student sample, which might be explained by the relatively low levels of aggression in this target group. Consequently, the app should also be investigated in populations with aggression regulation problems. Future research might also focus on the use of SCT to improve other types of behaviour driven by self-control, such as physical activity or smoking. Finally, a more personalized version of the app, in which users can select the number and types of SCT-tasks, should be developed and evaluated.

## Highlights

• We developed an app- and e-mail-based self-control training in which participants use their non-dominant hand for daily tasks.
• Self-control but not aggression improved in the group that received the app, and not in the e-mail based and control groups.
• A 2x2 full factorial design was used, with e-mail versus app, and five tasks at once versus one daily tasks as factors.

## 1 Introduction

### 1.1 Self-control and aggression

Self-control - the ability to prevent or override unwanted thoughts or behaviour (Muraven et al., 1999) - is a construct that is related to a broad range of behaviour, amongst which reactive aggression, academic success and physical health (Tangney et al., 2004). Despite its strong relationship with these behaviours, self-control is underrepresented in interventions used in clinical practice (Denson et al., 2012; Gottfredson and Hirschi, 1990). A type of behaviour that can benefit from more focus on self-control is reactive aggression, which refers to impulsive and uncontrolled outbursts of anger as a rection to a threat, provocation or frustration (Poulin and Boivin, 2000; Dodge and Coie, 1987). Reactive aggression is a complex societal problem that can take on many forms (Krug et al., 2002). Reactive aggression is associated with a broad range of personal and societal problems, such as violence against public servants, hooliganism, bullying in schools, bar fights, domestic violence, or violence within psychiatry (Geoffrion et al., 2017; Van Dijk et al., 2007; Dijk et al., 2007; Pekurinen et al., 2017; Rutherford et al., 2007). It is considered to be important, but also very challenging to reduce reactive aggression. The predominant treatment approach of aggression is based on the cognitive model (Polaschek et al., 2005; Ross et al., 2013). While treatment based on these models, such as cognitive behavioural therapy, has been helpful, meta-analyses show that effect sizes of these types of treatment for aggression lag somewhat behind on those of disorders such as anxiety and depression (Del Vecchio and O'Leary, 2004; Saini, 2009). Consequently, there is a need for more effective interventions that decrease reactive aggression (Ross et al., 2013; Gaynes et al., 2017). Interventions that target self-control seem to be a promising direction to achieve this.

### 1.2 Self-control training

An existing but underused intervention that has been shown to bolster self-control is self-control training (SCT; Friese et al., 2017, Beames et al., 2017, Hagger et al., 2010). SCT is a straightforward intervention in which participants are asked to perform tasks that require self-control. These tasks require them to override an impulse and replace it with a preferred response for a pre-specified period of time, often two weeks (Berkman, 2016; Friese et al., 2017). A well-studied form of SCT is the use of one's non-dominant hand for daily tasks such as brushing teeth, opening doors, or picking up items (Friese et al., 2017; Finkel et al., 2009). A proposed working mechanism is that participants practice in repressing an automatic response and replace it with a non-automatic response, such as using one's non-dominant hand, which - according to the strength model - improves the self-control ‘muscle’ (Baumeister et al., 2007). Strengthening this muscle is hypothesized to have a positive influence on behaviour that is driven by self-control, such as reactive aggression. (Friese et al., 2017; Beames et al., 2017; Hagger et al., 2010).

SCT has been studied in the context of physical activity, school performance or quitting smoking, but its application in treatment of aggression is lagging behind (Friese et al., 2017, Beames et al., 2017, Hagger et al., 2010). Two small experimental studies that did focus on aggression and applied the non-dominant hand paradigm showed promising results (Finkel et al., 2009; Denson et al., 2011). In the first study, 40 undergraduates were assigned to either SCT or a no intervention control condition for two weeks (Finkel et al., 2009). Self-control was first depleted via an attention control task. Results showed that participants reported a decrease in physical inclinations to harm their romantic partners in an experimental set-up, while no decreases in these aggressive inclinations of the control group were observed. In the second study, 70 undergraduates were included, and SCT was delivered to them in the same way as the previous study (Denson et al., 2011). Compared to a control group, participants that followed SCT were less aggressive than participants in the control condition, which was especially true for those high in trait aggressiveness. This was assessed in a lab by means of a task in which they were given the opportunity to retaliate an actor that insulted them by blasting loud blasts of white noise. While both studies showed the potential of SCT in increasing self-control and reducing aggression, more research is required to be able to draw more robust conclusions.

### 1.3 The potential of SCT for clinical practice

SCT has multiple advantages for clinical practice. Amongst other things, it does not require a high level of cognitive skills in its users because the tasks are straightforward, and SCT is easy to administer due to its simple instructions. Because of those characteristics, SCT seems to fit well with hard-to-reach target groups that, amongst other things, have difficulties with reflecting on their own behaviour due to externalization, such as blaming others for their aggression, and have cognitive deficits such as problems with memory and attention (Deenik et al., 2019; Drieschner and Boomsma, 2008). However, in existing studies, SCT is delivered face-to-face by researchers (Finkel et al., 2009; Denson et al., 2011). This implies that that if it is used in practice, healthcare professionals should deliver SCT to patients or other participants, taking up precious time that can also be used for other purposes. An app seems like a good solution to implement SCT in practice for multiple reasons. First, an app is scalable and easy to implement since it can be accessed by many people (van Gemert-Pijnen et al., 2018) without requiring sparse time from already overworked healthcare staff. Second, technology design principles, for example persuasive design, can be applied in order to increase adherence and engagement for people who are not that motivated to use SCT. An example of this is the forensic psychiatric patient population, whose treatment is often obligatory because they have committed an offense (Deenik et al., 2019; Drieschner and Boomsma, 2008; Kelders et al., 2012; Ludden et al., 2015). To illustrate, an app can send reminders to ensure that participants remember to do their SCT-tasks, and rewards can be added to keep them motivated. Since delivering SCT via an app is a novel approach, research is required to determine whether the use of an app is indeed of added value compared to delivering SCT via written instructions provided by a researcher.

### 1.4 The aim of this study

To summarize, there is an obvious need for more research into SCT. First of all, in order to draw more robust conclusions, there is an obvious need for more research on SCT and aggression (Finkel et al., 2009; Denson et al., 2011). Second, these types of evaluation studies should be conducted in the real world, assessing self-control during real-life instead of in a laboratory setting (Friese et al., 2017). Third, merely determining if SCT works does not suffice: research should determine what the most optimal way of delivering SCT is (Friese et al., 2017). For example, how many tasks of the non-dominant hand paradigm should be delivered to the participants? For example, is there a difference in effectiveness between one per day, or five tasks at once? And which tasks are most suitable for SCT? Fourth, while delivering SCT via an app seems to be a feasible, there is a need to investigate whether using an app actually is of added value compared to plain written instructions via for example e-mail. Before implementing SCT to increase self-control and reduce aggression, it is advisable to identify the most optimal way of delivering SCT. In order to answer these types of questions and to create the most optimal version of an SCT app, this study is focused on the evaluation and accompanying optimization of SCT to investigate the most effective and efficient way of delivering SCT. In order to achieve this, this study applies a 2 × 2 full factorial design with a control group. The main objective is to determine whether SCT is more effective for participants using an app- or e-mail-based version compared to a control group (factor 1), and whether it is more effective for groups that receive daily tasks or five tasks at once (factor 2), again compared to a control group. This goal is accompanied by the following research questions:

1. Are there differences between the improvements over time on self-control and aggression between the group that received SCT via an app, via e-mail, and the control group?
2. Are there differences between the improvements over time on self-control and aggression between the group that received one daily task, five tasks at once, and the control group?
3. What are the experiences with and points of improvement of the SCT intervention according to the participants?

## 2 Methods

### 2.1 Pilot study: design and outcomes

To lay the groundwork for this study, a pilot study was conducted (da Silva, 2019). The main goals were to compare the experiences of the participants with an app- and e-mail based version of SCT, and to identify points of improvements regarding the design and content of the SCT-app and methods used for the evaluation. Because the current study was based on this pilot study, its main methods and results will be briefly described here. Supplementary figures and tables about the results of the pilot study can be found in Appendix A.

In the pilot study, an SCT app was developed on a platform designated to develop apps for research (The Incredible Intervention Machine; TIIM), owned by the BMS Lab of the University of Twente. The instructions and non-dominant hand tasks were based on descriptions of the SCT of two previous studies that applied the non-dominant hand paradigm to reduce aggression (Finkel et al., 2009; Denson et al., 2011). The first version of the app, which was named Hands-on, was created by means of methods from human-centred design: paper prototyping, high-fidelity prototyping in the TIIM, and expert-based think-aloud usability tests with six experts on eHealth design of the University of Twente (Burns, 2018). Persuasive elements were added: reminders by sending notifications to the users twice a day to support them in remembering to perform the task; personalization by mentioning the user's name; and praise by complimenting users when they indicated they completed a task (Oinas-Kukkonen and Harjumaa, 2009). To investigate whether the use of the persuasive app to deliver SCT was of added value compared to a standard form of instruction, SCT was also delivered via e-mail, which contained similar instructions as the app, but no persuasive elements.

The overall goal of the pretest-posttest pilot study without control group was to identify whether there was a significant increase in participants' self-control and decrease in their aggression during and after the self-control training (SCT) using the non-dominant hand paradigm. In total, 19 university students were randomized into two different groups, with an ABA (*n* = 9) and BAB (*n* = 10) structure. In phase A, each day a new task was presented to the user via the app, and in phase B, one e-mail was sent with five tasks at once (Denson et al., 2011; Finkel et al., 2009). Each phase lasted 5 days, resulting in a total of 15 intervention days. Self-control and aggression of participants were assessed every five days by means of the Brief Self-Control Scale (BSCS; Tangney et al., 2004) and the Brief Aggression Questionnaire (BAQ; Webster et al., 2015). After completing the study, semi-structured interviews on the experiences with the app, points of improvement of the design and content of the app, and differences between the app and e-mail conditions were conducted with 10 randomly selected participants who completed the study. The goal of these interviews was to collect input for the further design of the SCT-app, again based on principles of human-centred design (Burns, 2018).

Repeated measures linear mixed models showed no interaction effects, but a main effect for time was found on both BSCS and BAQ scores, indicating that that self-control and aggression improved during and after the use of the SCT intervention. Additional analyses showed that, for Group 1 (app, e-mail, app), self-control and aggression did not improve over time, while this was the case for Group 2 (e-mail, app, e-mail). This result was unexpected because researchers found it more likely that the group who received SCT via app more often would have performed better. This showed the need for further investigation into the most optimal way of offering SCT. Furthermore, the interviews showed that participants considered the app easy to use and well designed. In general, participants preferred the app over the e-mail instructions (*n* = 8), mainly because the app sent them reminders to support the use of the non-dominant hand.

### 2.2 Design

**Fig. 1:**
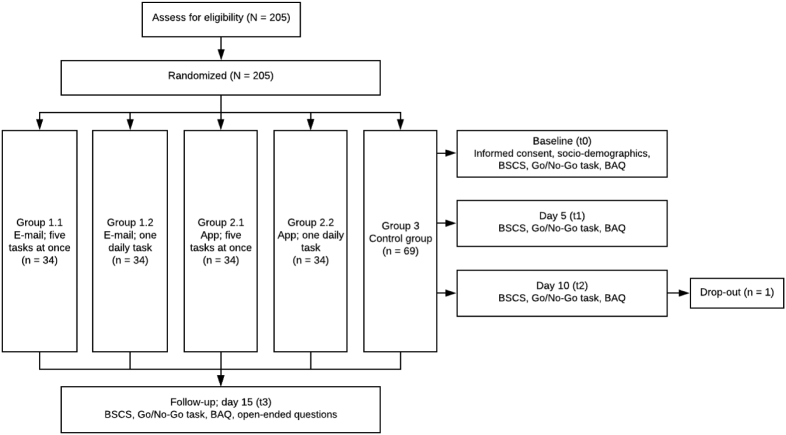
An overview of the 2 × 2 full factorial design of this study.

To answer the research questions, a 2 × 2 full factorial design was used to compare the effects of the app versus e-mail, and one daily task versus five tasks at once. As can be seen in Fig. 1, four experimental conditions and one control group were used. The levels of the factors were app versus e-mail, and daily tasks versus five tasks at once. In order to answer the third research question, all participants were asked to answer several open-ended questions in the final questionnaire at post-intervention assessment. This study was approved by the ethical committee of the University of Twente (application number 200019).

### 2.3 Participants

Because the focus of this study is on identifying the most optimal way of SCT, we evaluated the app with university students, who are easier to involve in research and for whom the underlying working mechanisms of SCT regarding self-control are expected to be the same as for other groups (De Ridder et al., 2012). Recruitment took place via Sona, a test subject pool of the University of Twente, via which students from social sciences can participate in studies in an exchange for course credits. The participants had to be at least 18 years old and had to be able to use the self-control training (SCT) app for 10 consecutive days. Participants were excluded if they were unable to use their hands for daily tasks or if they were ambidextrous. This convenience sample resulted in a total of 205 participants who started the study, of which 204 filled out the questionnaires in the follow-up measurement at t3 and were included in the analyses. However, not all participants filled out all questionnaires. Most of the participants were psychology students (85.80%), the remaining 27 students studied communication science (13.20%). The mean age of the 204 students who completed the follow-up was 20.33 (SD = 2.35), and 143 (70.10%) were female, 59 male (28.90%) and 2 preferred not to answer (1%). Most students (77.50%) were German, 16.70% was Dutch, and 5.90% had a different nationality. Finally, for most participants, the right hand was their dominant hand (89.70%).

### 2.4 Materials

#### 2.4.1 The HandSwitch app

**Fig. 2:**
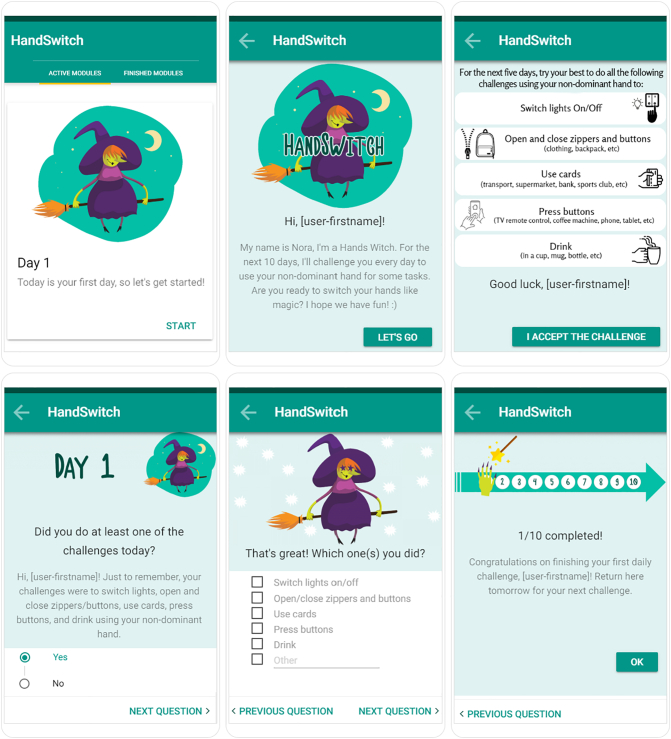
Screenshots of the five task version of the HandSwitch app.

Based on the outcomes of the pilot, several minor changes were made to the design and content of the app, see Fig. 2 for the version of the app that contains 5 tasks at once. This adapted version was again developed in TIIM. More attention was paid to the visual attractiveness of the design and two additional reminders a day were added (8 a.m. and 8 p.m.), and tasks that were too hard to execute based on the outcomes of the pilot were removed. Furthermore, because of the factorial design, two different versions of the app were created: one in which one daily task was provided, and one in which all five tasks were presented at once.

#### 2.4.2 E-mail self-control training

Due to the factorial design, two different e-mail conditions were used to investigate if there is a difference in effectiveness of SCT with one daily task or five at once. In one condition, participants received an e-mail with all five tasks at once. In the other condition, they received five daily e-mails, each containing a new task. As soon as the participant was allocated to one of the two conditions, the e-mails were scheduled to be sent automatically at the correct days and times. It was explicitly mentioned that, if necessary, participants could contact the researcher with questions about the intervention. Furthermore, several images that were used in the app were added to the e-mail as well, such as the hands-witch.

#### 2.4.3 Brief Self-Control Scale (BSCS)

In order to measure self-control, the Brief Self-Control Scale (BSCS; Tangney et al., 2004) was selected because it is a widely used and well-validated measure of self-control (Duckworth and Kern, 2011; Fung et al., 2019; Lindner et al., 2015). The BSCS is a brief self-report questionnaire that consists of 13 items (Tangney et al., 2004), which means that it is easily administrable. The BSCS measures trait self-control via items with a 5-point Likert scale, where 1 refers to ‘not at all’ and 5 to ‘extremely’, so higher scores represent higher levels of self-control. Examples of items are ‘I am good at resisting temptation’ and ‘Pleasure and fun sometimes keep me from getting work done’. In the pilot study, the Cronbach's alpha of the BSCS at the five measuring moments ranged between 0.786 and 0.911, showing an overall high reliability (Da Silva, 2019). In the present study, the Cronbach's alpha of the four measurements of the BSCS ranged between 0.790 and 0.852, showing a good internal consistency at all measuring moments and is comparable to the original BSCS Cronbach's alpha values: 0.83 and 0.85 (Tangney et al., 2004). At t1, the original version of the scale was used while at t2, t3 and t4 slight adaptations in phrasing were added to ensure that the BSCS covered self-control over the past five days.

#### 2.4.4 Brief aggression questionnaire (BAQ)

Aggression was measured by the Brief Aggression Questionnaire (BAQ), a 12-item validated scale to measure trait aggression (Webster et al., 2015). The BAQ measures four dimensions: physical aggression, anger, verbal aggression, and hostility. Examples of items are ‘I have trouble controlling my temper’ and ‘Given enough provocation, I could hit another person’. Participants score the extent to which an item applies to them on a 5-point Likert scale, where 1 represents ‘extremely uncharacteristic of me’ and 5 ‘extremely characteristic of me’, meaning that higher scores represent higher levels of aggression. The BAQ was selected because it showed good reliability in the pilot: the alpha ranged between 0.712 and 0.894 at the five measurement points, which is comparable to the alpha of 0.79 that was identified in earlier research (Webster et al., 2015). In order to gain insight into changes over time, the BAQ at t1, t2 and t3 was slightly adapted to cover only the past five days instead of the past year. In this study, the Cronbach's alpha of all four BAQ measurements ranged between 0.661 and 0.773. Only at t0 the alpha of 0.661 was considered low, while at the other three moments it can be considered as good.

#### 2.4.5 Go/no-go task

Because a large meta-analysis on self-control measures indicated that self-control is best assessed using multiple methods because it is a complex, multidimensional construct, we also used a different type measure for self-control, besides the self-report scale (Duckworth and Kern, 2011). In line with these recommendations, self-control was also measured with an executive function task. The Go/No-Go task is a well-studied measure of cognitive control and has been used in previous research to assess self-control (Duckworth and Kern, 2011). In the Go/No-Go task, participants are instructed to respond to target stimuli, but have to refrain from responding to non-target stimuli, which requires suppressing a behavioural response. In the current study, the average reaction time was calculated, in which shorter reaction times are expected to represent higher levels self-control.

#### 2.4.6 Experiences and subjective task performance

To gain insight into the experiences of the participants that received the app or e-mail, three open-ended questions were asked in the final assessment at t3. Participants were asked (1) to explain whether they experienced any effects on self-control due to the intervention, (2) what their opinion of the intervention was, and (3) what points of improvements for the content and design of the app or e-mail they identified. Furthermore, participants indicated for all 10 SCT-tasks whether they should be removed and - if they wished to remove it - why this was the case. For each task, they were also asked to rate on a 5-point Likert scale how well they believe they performed on the task and how difficult it was, where 1 represented very bad and very difficult respectively, and 5 very good and very easy. Finally, participants were asked to suggest other tasks that could be incorporated in the intervention.

### 2.5 Procedure

Participants were informed about the goal of the study in general terms; self-control was not explicitly mentioned to prevent bias. As can be seen in Fig. 5, after providing informed consent, participants were assessed four times: once directly before starting with SCT (t0), twice during SCT (t1 & t2), and once five days after completing SCT (t3). At all measuring points, the BSCS, Go/No-Go task and BAQ were completed via Qualtrics. Filling out the questionnaires took on average 15 min. Also, app users were asked to indicate their subjective performance and difficulty of tasks in the app, while participants who received e-mails were asked to rate this via Qualtrics. Participants in the control conditions only received e-mails with an invitation to fill out the questionnaires. In the final questionnaire at t3, participants in the e-mail and app conditions were asked to answer three open-ended questions on the intervention in Qualtrics.

### 2.6 Analysis

An a priori power analysis was conducted with G*Power, with a medium effect size (d = 0.40), a β-power of 0.8, an alpha of 0.05 and an independent two-sided *t*-test to evaluate differences on self-control; the main outcome. Outcomes of the power analysis indicated that a total sample of 176 participants was required, with each main condition, for example the group that received SCT via an app, consisting of 59 participants. Accounting for a drop-out of 15%, a total sample size of 202 was required. Data were analysed using IBM SPSS software (version 24.0) and significance was accepted at 0.05 or lower. In order to check for differences at baseline on outcomes of the three measures, one-way between-subjects ANOVAs were used.

Due to the full factorial design of this study, the same analyses were conducted twice: once comparing the control group with participants receiving the SCT via app versus the participants that received SCT via e-mail, and once comparing the control group with the participants that received 1 task per day or 5 tasks at once. To account for autocorrelation within participants, repeated measures linear mixed models were used. Compound symmetry was used as the repeated covariance structure as this structure showed the best fit for the data across different models. Scores on the BSCS and BAQ and average reaction times of the Go/No-Go tasks on t0, t1, t2 and t3 were used as the dependent variables. Time and group were used as fixed factors, along with their interactions, and participants were modelled as random factor. Interaction effects showed whether the changes over time differed between groups, and main effects for time showed whether the scores of all participants changed over time. In order to provide more insight into the main findings, Least-significant difference (LSD) post-hoc analyses were run to further investigate potential significant differences between the three groups and changes over time per group. To investigate the effects of SCT on self-control and aggression for each separate group, repeated measures linear mixed models with time as the only fixed factor were used for each group, again using compound symmetry as the repeated covariance structure.

In order to answer the third research question on points of improvement, the written answers to the open-ended questions were analysed inductively by two researchers (HK & MdS), using the method of constant comparison (Boeije, 2002). Descriptive statistics were used to provide an overview of how many participants wanted to remove a task from the intervention.

## 3 Results

### 3.1 Descriptive statistics and baseline assessment

In Table 1 the average scores and standard deviations for all groups are provided for the three measures. No significant differences on baseline (t0) of the scores of the BSCS (*F*[2201] = 1.359, *p* = .259) and BAQ [F(2, 199) = 0.070, *p* = .932] were found for app, e-mail and control group, meaning that all groups showed comparable baseline levels of self-control and aggression. A significant difference was found on the baseline scores of the mean reaction times of the Go/No-Go task (*F*[2,202] = 3.283, *p* = .04). LSD post hoc analyses showed that the mean difference of the scores of the app group in milliseconds was significantly lower than that of the e-mail group (*M* = −44.26 [CI95–78.37, −10.13], *p* = .011). However, the used repeated measure linear mixed model corrects for these differences at baseline by using the group to which the participants were assigned as a fixed factor. Furthermore, for the 1 task, 5 tasks and control group, no significant differences on baseline (t0) were found for the scores on the BSCS (*F*[2, 202] = 1.417, *p* = .245); the average reaction times on the Go/No-Go task (F[2, 202] = 0.064, *p* = .938); and the BAQ scores (*F*[2,200] = 0.010, *p* = .990). This shows that these groups had similar baseline levels of self-control and aggression.

**Table 1:** Descriptive statistics of the scores on the Brief Self-Control Scale (BSCS), the Go/No-Go task and Brief Aggression Questionnaire (BAQ) for all groups on all measurement moments.

Main group	n	t0 (M; SD)	t1 (M; SD)	t2 (M; SD)	t3 (M; SD)	Difference t3 – t0 (M; SD)
Brief Self-control Scale (BSCS)						
E-mail	68	41.38; 6.32	41.71; 6.98	42.04; 7.73	41.62; 6.77	0.24; 6.17
App	67	41.78; 8.22	42.85; 6.93	44.07; 8.04	44.18; 7.92	2.39; 6.33
5 tasks	66	42.12; 6.99	42.70; 6.49	43.33; 8.27	43.41; 7.32	1.28; 6.23
1 task	68	41.07; 7.73	41.75; 7.46	42.69; 7.65	42.37; 7.65	1.29; 6.51
Control	69	40.07; 6.76	39.68; 7.06	40.56; 7.93	41.51; 7.51	1.43; 6.81
Total	204	41.07; 7.15	41.40; 7.08	42.21; 8.0	42.42; 7.51	1.35; 6.47

Go/No-Go task (in milliseconds)						
E-mail	68	241.56; 115.48	202.59; 112.68	165.76; 76.24	166.64; 102.50	−74.92; 107.40
App	67	197.30; 89.07	143.33; 70.60	149.55; 78.71	138.25; 79.31	−57.09; 82.79
5 tasks	65	221.83; 96.35	185.36; 98.90	197.06; 112.46	167.93; 109.16	−58.45; 95.23
1 task	68	216.64; 103.76	156.68; 82.64	150.22; 79.70	143.12; 91.32	−73.52; 97.73
Control	69	221.83; 96.35	185.36; 98.90	197.06; 112.46	167.92; 109.16	−53.91; 109.05
Total	204	220.24; 102.02	177.13; 98.42	171.02; 92.53	157.75; 98.44	−61.96; 100.52

Brief Aggression Questionnaire (BAQ)						
E-mail	68	30.19; 5.60	26.31; 6.08	26.28; 6.27	25.78; 6.29	−4.41; 6.53
App	67	29.99; 5.27	27.79; 5.32	26.75; 5.92	26.45; 5.66	−3.64; 4.94
5 tasks	65	29.94; 5.66	27.52; 5.82	27.38; 6.58	26.80; 6.10	−3.22; 6.03
1 task	65	30.25; 5.29	26.71; 5.71	25.87; 5.46	25.67; 5.77	−4.63; 5.43
Control	69	30.12; 5.87	26.33; 6.67	26.75; 6.77	27.22; 7.29	−2.88; 6.32
Total	204	30.10; 5.56	26.81; 6.06	26.59; 6.30	26.49; 6.45	−3.64; 5.98

### 3.2 App versus e-mail versus control group

#### 3.2.1 Self-control: BSCS

**Fig. 3:**
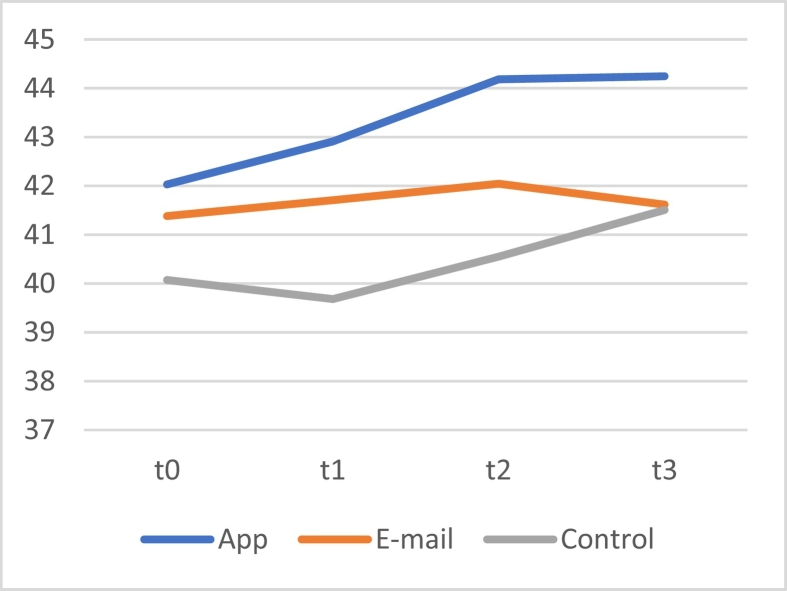
The means of the score on the BSCS of the app, e-mail and control group at the four measurement moments.

A linear mixed model for repeated measures showed no significant overall interaction effects between time and group (*F*[2, 807.713] = 8.75, *p* = .417). However, a significant main effect of time was observed (*F*[1, 807.714] = 4.525, *p* = .034), which shows that for all participants, the BSCS scores improved over time. No significant main effect of group was found (*F*(2, 597.578) = 1.027, *p* = .359), meaning that there were no significant differences between all BSCS scores between all three groups. Post hoc LSD tests showed a significant difference between self-control scores of the app and control group. As can be seen in the mean difference between groups, the scores of participants in the app group were higher (*M* = 2.627 [95CI +0.689, +4.566], *p* = .008). This shows that all self-control scores of the app group were on average higher than those of the control group. To illustrate these findings, a plot of the means of all three conditions was created (see Fig. 3). To further explore differences within the three groups, additional post hoc analyses were performed.

To zoom in on the changes over time within the three conditions, a linear mixed model using compound symmetry with only time as a fixed factor was used for each group separately. For the app condition, significant improvements in self-control over time were identified by a main effect of time (*F*[3, 196.315] = 4.090, *p* = .008). However, for the e-mail condition (*F*[3,201,000] = 0.251, *p* = .861) and the control group (*F*[3, 210.000] = 1.953, *p* = .122), no significant differences between scores over time were found. These results show that only in the app condition, self-control increased significantly over time.

#### 3.2.2 Self-control: Go/No-Go

**Fig. 4:**
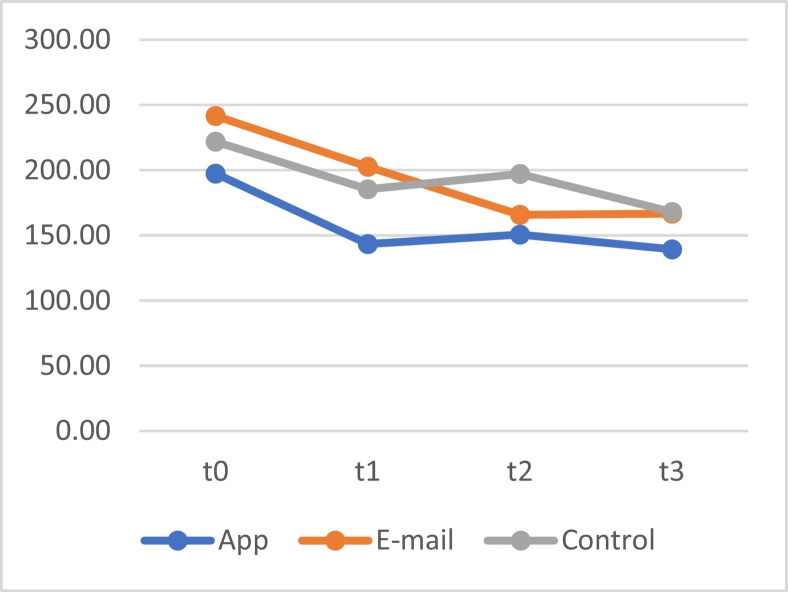
The mean reaction time for the Go/No-Go tasks in milliseconds for the app, e-mail and control groups.

A significant interaction effect was observed for the reaction times on the Go/No-Go task over time for all three groups (*F*[6, 604.172] = 2.630, *p* = .016), meaning that changes in reaction time differed between the three groups over time. As can be seen in Fig. 4, the reaction time of the control group increased at t2, while the score of the e-mail group, which was higher at t1, is lower at t2, which explains the interaction effect. A main effect for time was identified (*F*[3, 604.174] = 33.178, *p* < .001), meaning that all reaction times significantly decreased over time. Also, a significant main effect was identified for groups (*F*[2, 201,854] = 4.944, *p* = .008), which means that there was a difference between the groups in reaction time. LSD post hoc analyses show that the reaction times of the app group for all measurements were lower than those of the control group (*M* = −35.42 [CI −61.41, − 9.44], *p* = .008) and the e-mail group (*M* = −36.51 [CI −62.59, −10.44], *p* = .006).

To identify whether significant decreases in reaction time took place in each separate group, three linear mixed model analyses with only time as a fixed factor were conducted. A main effect for time was found in the app group (*F*[3, 198,984] = 16.391, *p* < .001), the e-mail group (*F*[3, 201,000] = 14.322, *p* < .001) and the control group (*F*[3, 204,000] = 8.188, *p* < .001). In line with Fig. 8, LSD post hoc analyses showed that for all groups, the decreases between the baseline measure at t0 and the three following measures (t1, t2 and t3) were significant (*p*-values ranged between <0.001 and 0.004).

#### 3.2.3 Aggression – app, e-mail and control group

**Fig. 5:**
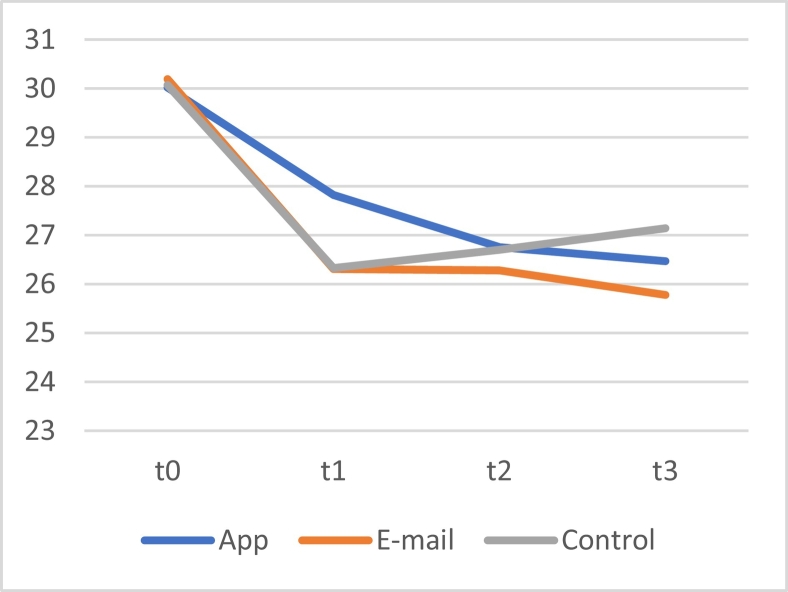
The means of the score on the BAQ of the app, e-mail and control group at the four measurement moments.

A repeated measures linear mixed model using BAQ scores showed no significant interaction effects between time and all three groups, (*F*[6, 602.594] = 1.525, *p* = .168). A significant main effect of time was observed (*F*[3, 602.597] = 46.663, *p* < .001), showing that the self-reported aggression of all groups decreased over time. The LSD post-hoc analyses showed no significant differences between the scores of all groups. As can be seen in Fig. 5, the BAQ scores on t0 seem to be substantially higher than the other three measuring moments. Additional analyses were conducted to investigate changes within and between groups.

To investigate whether this difference over time was significant for each separate group, a linear mixed model with only time as a fixed factor was used for each group. For all three conditions, respectively app (*F*[3, 195,447] = 14.753, *p* < .001), e-mail (*F*[3, 201,000] = 19.267; *p* < .001) and control condition (*F*[3, 209.120] = 16.318, *p* < .001), significant changes over time were observed. In line with Fig. 5, post-hoc LSD analyses showed significant differences (*p*-values all <0.001) between t0 and the remaining three measurement moments (t1, t2 and t3) for all three groups, indicating that scores remained relatively stable after the drop between t0 and t1.

### 3.3 5 tasks versus 1 task versus control group

#### 3.3.1 Self-control: BSCS

**Fig. 6:**
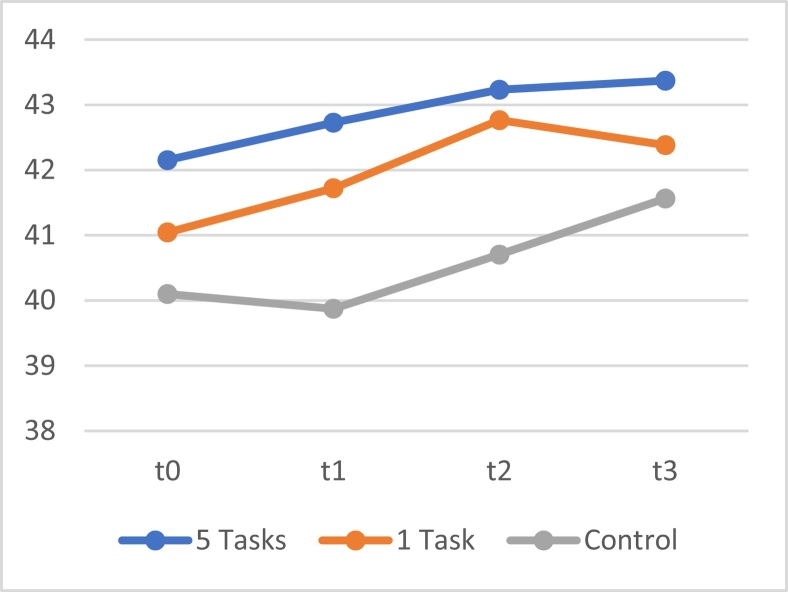
The means of the score on the BSCS of the group that received 5 tasks at once, the group that received one daily task, and the control group.

A repeated measure linear mixed model showed no significant interaction effects between time and all three groups on self-control (*F*[6, 604.393] = 0.400, *p* = .879). A significant main effect was identified for time (*F*[3, 604.397] = 4.109, *p* = .007), so for all participants, self-control increased over time. Post hoc analyses showed a significant difference between the means of the group that received 5 tasks at once and the control group (*M* = 2.311, [CI95 + 0.183, +4.438], *p* = .033). The average BSCS scores for all three groups are plotted in Fig. 6.

To further explore the results, a linear mixed model with only time as a fixed factor was used for each group separately. No significant effects of time were found for the group that received 5 tasks at once (*F*[3, 193.418] = 0.937, *p* = .424), the group that received 1 task per day, (*F*[3, 201.000] = 2.124, *p* = .098), and the control group (*F*[3, 210.000] = 1.953, *p* = .122). This means that the improvement of self-control within each separate group was not significant.

#### 3.3.2 Self-control: Go/No-Go

**Fig. 7:**
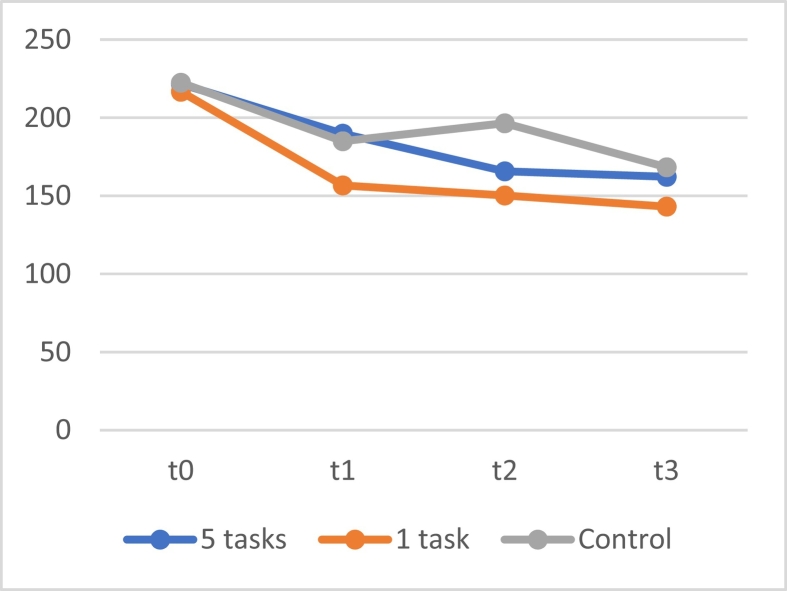
The mean reaction time for the Go/No-Go tasks in milliseconds for the group that received 5 tasks at once, the group that received one daily task, and the control group.

No significant interaction effect between time and group was found (*F*[6, 604.403] = 1.661, *p* = .128). A main effect for time was found (*F*[3, 604.409] = 32.967, *p* < .001), showing that reaction time for all participants significantly decreased over time. LSD post hoc analyses showed that the mean reaction times of the group that received 1 task were overall lower than those of the control group (*M* = −26.36 [CI95–52.51, −0.22], *p* = .048), which can also be observed in Fig. 7.

To further investigate the effect of time for the three separate groups, repeated measures linear mixed model with only time as a fixed factor were used for each group. A significant main effect of time was found for the group that received 5 tasks at once (*F*[3, 193.471]3 = 8.885, *p* < .001), 1 task per day (*F*[3, 201,000] = 20.330, *p <* .001) and the control group (*F*[3,210,000] = 8.614, *p* < .001). Post hoc analyses showed that for all groups, the decrease between t0 and the next three measures (t1, t2 and t3) were all significant, and all had a significance level of *p* < .001.

#### 3.3.3 Aggression – 5 tasks, 1 task and control group

**Fig. 8:**
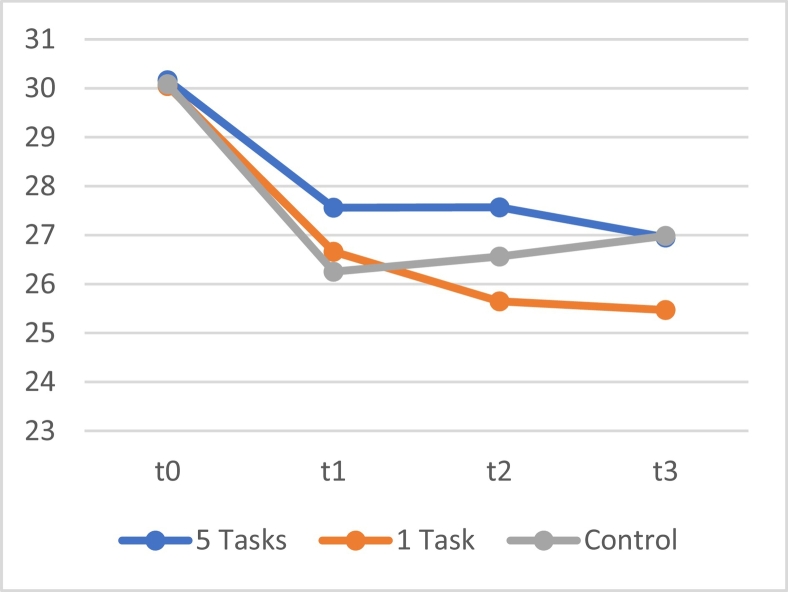
The means of the score on the BAQ of the group that received 5 tasks at once, the group that received one daily task, and the control group.

For the scores on the BAQ, no significant interaction effect between time and group was found (*F*[6, 602.592] = 1.551, *p* = .159). Post hoc tests showed no differences between groups. A significant main effect of time was found (*F*[3, 602.595] = 46.579, *p* < .001), indicating that the scores for all groups together decreased over time, as can also be seen in Fig. 8.

Linear mixed models for each separate group with only time as a fixed factor showed a significant main effect of time for the group that received 5 tasks at once (*F*[3, 193.338] = 9.305, *p* < .001), the group that received 1 task per day (*F*[3, 200.151] = 25.834, *p* < .001), and the control group (*F*[3, 209.120] = 16.318, *p* < .001). As can also be observed in Fig. 8, post hoc analyses again showed that these decreases were only significant (*p* values all <0.001) between t0 and the other three measurement moments.

### 3.4 Experiences and points of improvement

The codes show that most participants did not have any points of improvements and were satisfied with the intervention. The full coding schemes can be found in Appendix B, Appendix C. The most-mentioned point of improvement was to add more reminders since participants indicated they often forgot about the tasks during the day. Also, participants indicated that some tasks were too difficult and needed to be either removed or adapted. As can be seen in Appendix D, almost two thirds (62.22%) of the participants indicated that task 10 (writing with the non-dominant hand) should be removed, mostly due to its negative impact on daily life, such as making notes during lectures or signing official documents. For the other nine tasks, less than a quarter (range of 1.48% to 24.44%) of the participants indicated that it should be removed.

Another point that arose from the answers was that several participants experienced minor bugs in answering questions about how well the task went in the app. They noted that this did not affect the intervention itself. Also, some participants who received five tasks at once indicated that they would have preferred to receive less tasks, while several participants who received a daily task would rather have received more tasks at once. Furthermore, multiple other recommendations were made by relatively few participants, for example, four participants expressed the need to know more about the reason for using one's non-dominant hand since they did not see the relevance. Finally, 24 participants had suggestions that did not pertain to the intervention, but targeted the study design, e.g. remarks on the used questionnaires.

## 4 Discussion

### 4.1 Summary and interpretation of results

The goal of this study was to provide insight into the effectiveness and points of improvement of a self-control training (SCT) intervention to increase self-control and reduce aggression. The first research question aimed to determine if an app-based version of SCT was more effective than an e-mail based version in improving self-control and decreasing aggression. While no interaction effects were found, analyses showed that only self-reported self-control of participants that used the app improved over time; no improvements over time were observed in the e-mail and control condition. Furthermore, while participants that used the app performed overall better on the Go/No-Go tasks, there was no interaction effect, which means that the differences might be explained by existing differences at baseline. Additionally, no meaningful differences between groups were found on self-reported aggression. The second research question was focused on investigating whether the number of tasks is related to effectiveness. No significant differences between groups were found, which implies that the number of tasks is not a component that adds to the effectiveness of SCT. The third research question was focused on the experiences of the participants with the app- and e-mail-based version of SCT. The qualitative results showed that participants were satisfied with the design and content of both app and e-mail SCT, but they preferred to receive more reminders to perform the non-dominant hand tasks. Overall, self-control but not aggression improved in the group that received app-based SCT, and not in the e-mail based and control groups, y. Regardless, more research is required to further investigate the effectiveness of the SCT app.

### 4.2 Personalization of the SCT app

Our findings and earlier research into SCT show the potential that a mobile app might have for the delivery of SCT compared to e-mail (Finkel et al., 2009; Denson et al., 2011; Friese et al., 2017; Beames et al., 2017). While further research into its effectiveness is essential, it is recommended to further improve and investigate the SCT-app. The increase in self-control over time of the participants that received the app was quite small, indicating that there is room for improvement. The quantitative and qualitative results of our study showed multiple ways to further improve the app. However, based on the outcomes, there does not seem to be one way to improve the app for all users, which points into the direction of creating multiple versions of the app. For example, several participants who received one task indicated that they would have preferred to receive more tasks at once, and vice versa. This shows that there are differences in pretences, which poses an opportunity for personalization. Because analyses showed that the number of tasks is not a component that adds to the effectiveness of SCT, this can be personalized. Another point of improvement was related to the suitability of the tasks of SCT. The participants' ratings per task showed that there was difference between their opinions and experiences. Finally, since not all users seemed to enjoy the hand-switching tasks or indicated that they might get bored on the long term, other types of SCT-tasks can be added to keep users engaged. An example of a different type of SCT-task is refraining from using common slang words (Finkel et al., 2009). An advantage specific for technology-based interventions is that users can individually compile their own intervention, and thus select the content and number of tasks to their preferences (Andersson et al., 2011; Brouwer et al., 2011; Ludden et al., 2015). By creating such a personalized version of the SCT-app, users are offered the best fitting opportunity to train their self-control.

### 4.3 Design of the SCT app

A main advantage of eHealth compared to in-person care is that it has the potential to increase user engagement with and adherence to an intervention, which in its turn can improve its effectiveness (Ludden et al., 2015). In our study, participants appreciated the app and were positive about its usability and visual design. This positive assessment can partly be explained by the involvement of users and experts in the pilot when designing the app. These types participatory development methods can lead to a better fit between the technology and the needs and wishes of the user (van Gemert-Pijnen et al., 2011; Michie et al., 2017). In line with this presumption, almost all participants in this study indicated that they used the app for 10 days. However, it might be more challenging to engage hard-to-reach target groups such as prisoners or forensic psychiatric patients who committed violent crimes. Consequently, a next version of the app could benefit more from the possibilities of technology to further engage users. A possible way to achieve this is by means of gamification, in which elements from game-design such as social comparison, mastery or rewards are added (Sailer et al., 2014). Furthermore, because adding persuasive features - such as reminders, praise or rewards - can lead to increased adherence, more of these features can be added to SCT app (Kelders et al., 2012; Ludden et al., 2015; Oinas-Kukkonen and Harjumaa, 2009). An especially important feature for the SCT app seems to be reminders. To illustrate: participants in the e-mail condition did not receive daily reminders, which can explain why no significant improvements in self-control over time were identified for these participants. Also, even though app-users received reminders, this was not perceived as enough by various participants. However, since reminders can be experienced as annoying by users (Westermann et al., 2015), participants should be offered the option to adapt the number, timing and content of reminders to their own preferences, again highlighting the importance of a personalized SCT app.

### 4.4 Strengths & limitations

No interaction effects were found in this study. When calculating the power, we assumed a medium effect size, but since no comparable studies were available on which we could base the effect size, this estimation might have been too high. Regardless, we were still able to gain some insights into effectiveness of the app. A main limitation of this study was the measurement of aggression. The results showed a strong drop in average BAQ-scores after the baseline measure and a low Cronbach's alpha of the BAQ on baseline. This can be explained by the way questions were phrased: at the baseline measurement, the BAQ focused on aggression in general, and after that, the focus was on the past five days, which might have influenced the scores. However, the repeated measures linear mixed models we used accounted for these differences and showed no differences in changes in aggression between groups, which means that this measurement error did not affect the conclusions of this study. Finally, the rewards that were received by participants might have influenced the validity of the results: participating students received credits for their education if they finished the study and thus had to use the app for 10 days. This situation is not representative for real-life, in which more users might drop out due to a lack of rewards: non-adherence is indeed a big issue for eHealth interventions (Brown et al., 2016). Regardless, participants indicated that they liked the intervention and reminders were included, which might positively impact adherence in real life.

### 4.5 Future research

#### 4.5.1 Evaluation

Since this is, to the best of our knowledge, the first study that evaluated a self-control training app, we aimed to not just gain insight into if the app works, but also into which elements contributed to the effectiveness by means of a 2 × 2 full factorial design. While promising results were found on the potential of the SCT-app to improve self-control over time, additional research is needed to further investigate and replicate these findings. Based on our results, a personalized SCT-app should be developed. This might raise questions about how to evaluate the effectiveness of all of these different versions of a personalized app. However, while the content and design of these versions might not be completely identical, the underlying intervention principles are the same (Mohr et al., 2015). Consequently, in order to establish whether a personalized version of the app is more effective than a ‘locked-down’ version, a three-armed randomized controlled trial can be conducted, according to the ‘trial of intervention principles’ (Mohr et al., 2015). Additionally, to identify which components of an improved version - such as reminders or gamification - add to the effectiveness, other fractional or full factorial designs can be employed (Collins et al., 2014; Collins and Kugler, 2018).

Besides gaining more insight into how the design of the app can contribute to effectiveness, more insight into how SCT works is required as well. For example, what are the crucial elements that make SCT effective (Friese et al., 2017)? While this study already provided some answers, there are more questions that need to be addressed. For example, more insight is needed into how long the effects of SCT remain, since they might wash out fairly quickly after finishing the intervention. Not many studies on SCT used a follow-up measure, and our only follow-up was merely five days after completing the intervention (Friese et al., 2017). In future research, more follow-up measures should be used over a longer period of time to gain insight into how long the effects of the SCT app remain.

Also, it might be interesting to determine for how long SCT should ideally be administered (Friese et al., 2017). An example of a suitable evaluation method for these types of questions is an introduction/withdrawal single-case experimental design (Krasny-Pacini and Evans, 2018; Dallery et al., 2013). Future research should apply fitting and innovative research designs to further open the black box of the SCT app.

#### 4.5.2 New applications of SCT

In this study, students participated since this target group is easy to involve in research, and working mechanisms behind SCT to bolster self-control are expected to be the same (Friese et al., 2017). Our findings regarding self-control were fairly promising, but we found no interaction effects. An explanation for this is that in our student sample, self-control on baseline was already relatively high, and aggression relatively low, leaving not much room for improvement. Other target groups with more self-regulation problems might benefit more from the SCT-app., like forensic psychiatric patients, delinquent youth or prisoners. Since assessing aggression via self-report measures might be prone to multiple types of biases such as memory or social desirability bias, they can be combined with other types of measures, such as reports or questionnaires on aggressive behaviour filled out by staff (Kobes et al., 2012). Regardless, measuring aggression is a difficult task, highlighting the need for future research. Also, measures that do not require reading, such as the Go/No-Go task that was used in this study, might be very suitable for these types of target groups, who often are semi-illiterate or have other cognitive deficits (Greenberg et al., 2007; Clausen et al., 2016). Further research is needed to ensure the suitability and validity of these types of measures for evaluating the SCT-app.

SCT cannot just be applied to different target groups, but also to other target behaviours. Many existing (eHealth) interventions that aim to improve goal-driven behaviour such as a lack of physical activity, smoking or unhealthy eating, suffer from the intention-behaviour gap (Sniehotta et al., 2014). This means that participants have trouble sticking to their goals, which might be explained by a lack of self-control (Pfeffer and Strobach, 2017; Sniehotta et al., 2005). Therefore, SCT can be used as an addition to these types of interventions to bridge the intention-behaviour gap and thus increase their effectiveness. SCT has indeed been shown to improve a broad range of health-related behaviours (Tangney et al., 2004), so it would be interesting to study the potential of an SCT app to bolster the effectiveness of existing, goal-driven eHealth interventions in a relatively cheap, scalable and easy way.

## 5 Conclusion

This study showed that self-control of students who used self-control training (SCT) app increased over time, as opposed to a group that received SCT via e-mail and a control-group. No effects on aggression were found, which might be explained by the limitations of the measure used or the extent to which aggression was a problem of the target group. Based on our results, future research in which the SCT app is further improved and evaluated is warranted, since it might have the potential to increase self-control. Based on the findings of this study, an improved, personalized version of the app might be developed, in which content and design could be adapted to fit individual users. Future research should provide more insight into if and how SCT works, and how the possibilities of mobile apps can be used to further bolster its effectiveness.

## Declaration of competing interest

This study was funded by Stichting Vrienden van Oldenkotte. Funding for this study was provided by Stichting Vrienden van Oldenkotte. They had no role in the study design, collection, analysis or interpretation of the data, writing the manuscript, or the decision to submit the paper for publication. All authors declare that they have no conflicts of interest.

## Figures and Tables

**Fig. A1:**
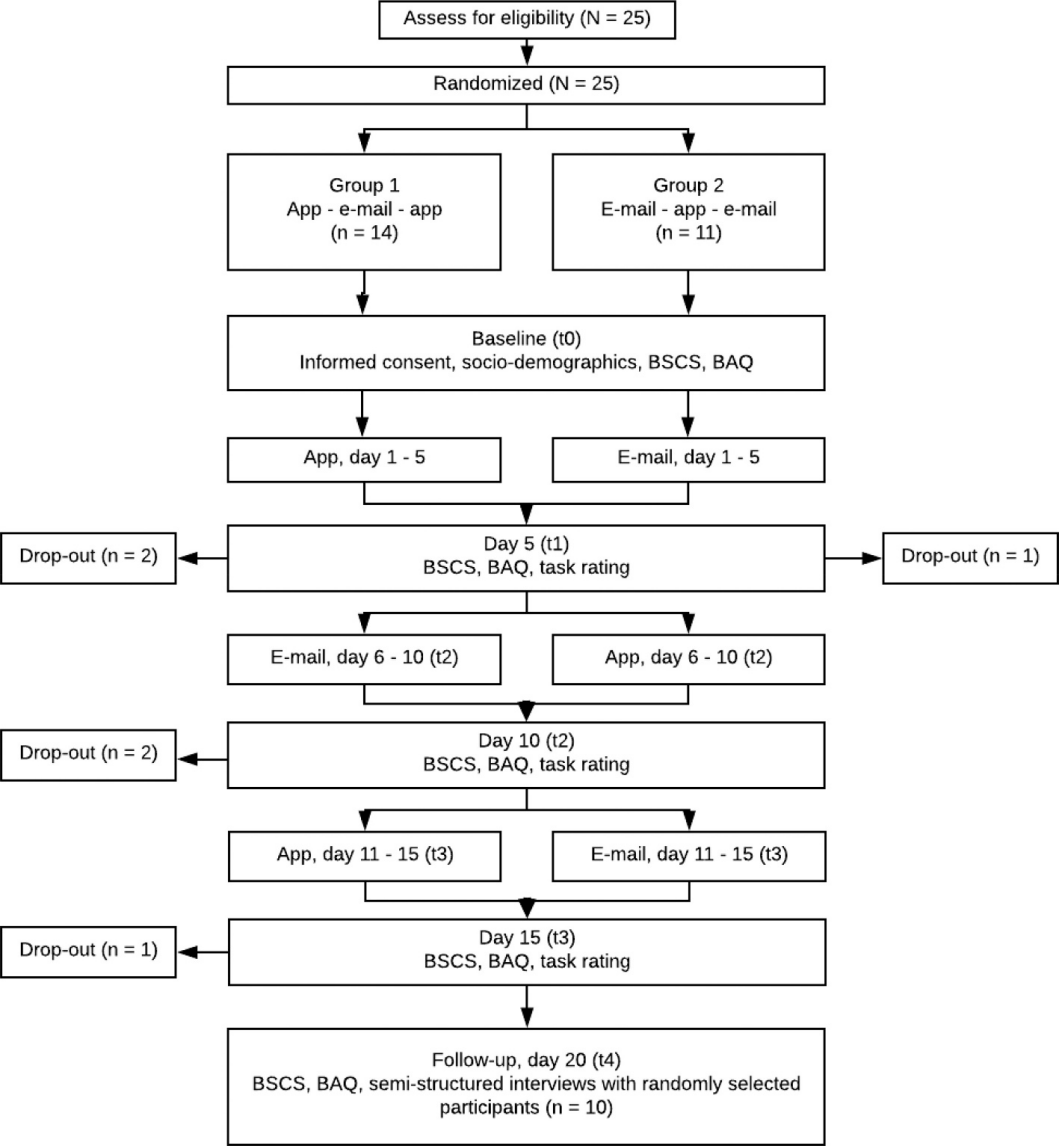
Overview of the pretest-posttest study design of the pilot study.

**Fig. A2:**
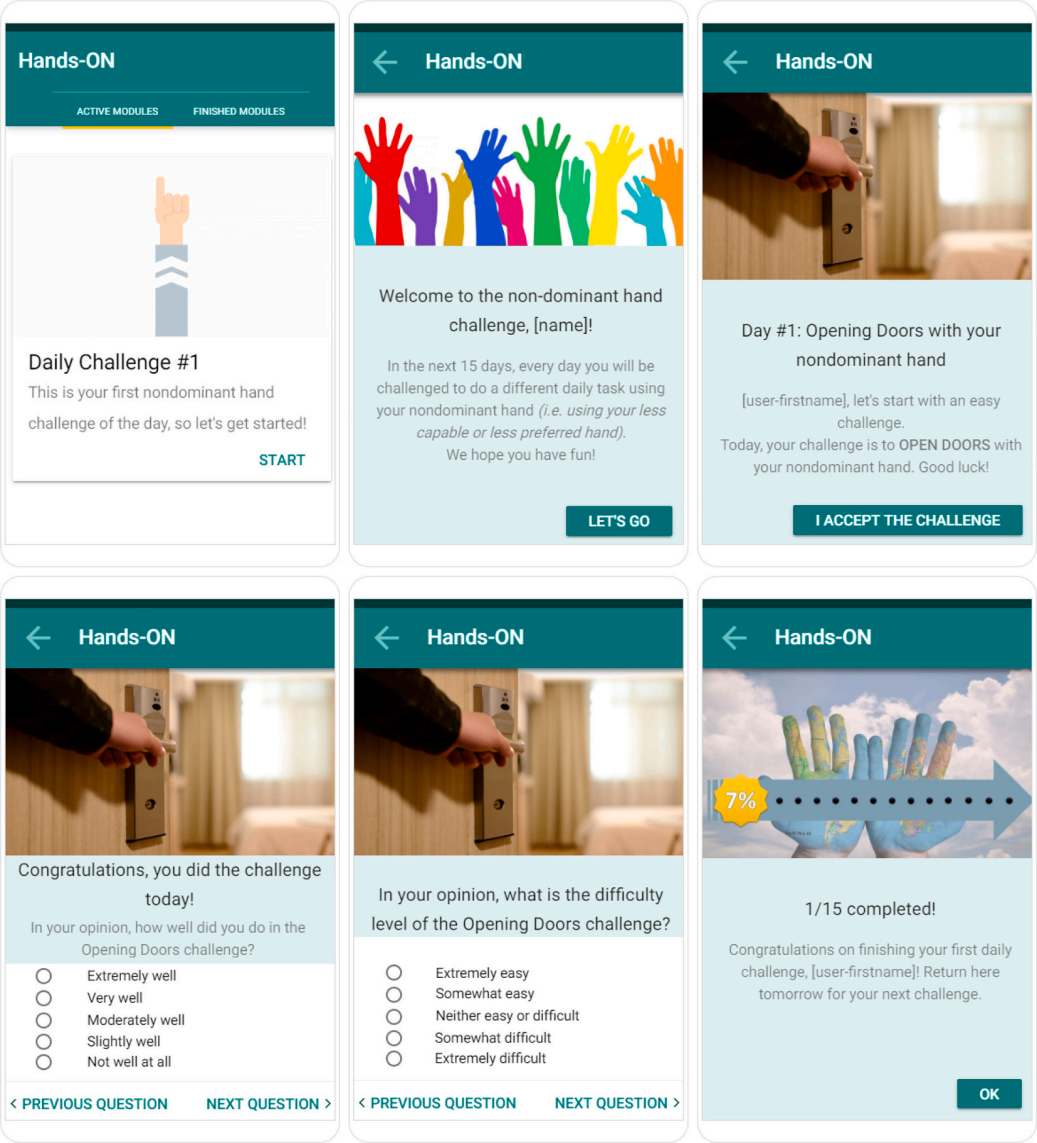
Screenshots of the Hands-ON app in which one task per day is provided, developed in The Incredible Intervention Machine (TIIM) application of the University of Twente.

**Fig. A3:**
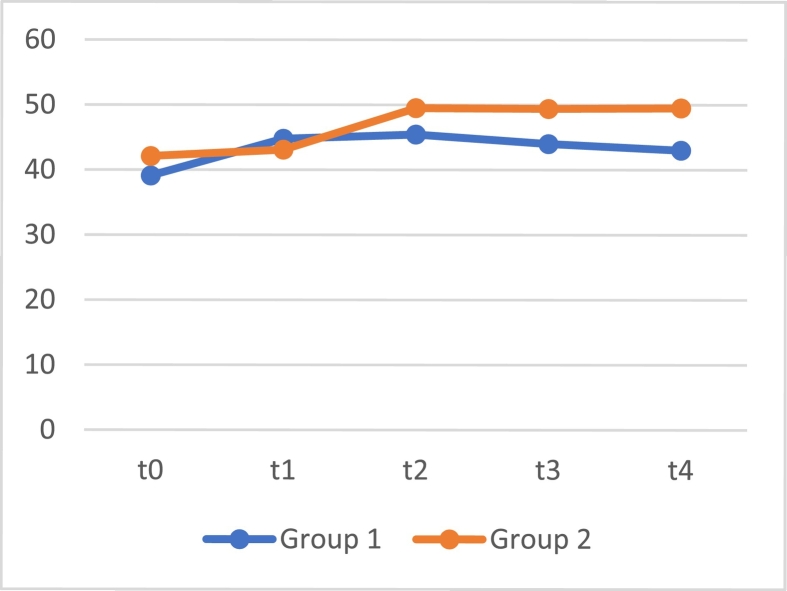
The BSCS scores of Group 1 (app, e-mail, app) and Group 2 (e-mail, app, e-mail) over time, with a main effect for time (*F*[4, 68,000] = 5.606, *p* = .001).

**Fig. A4:**
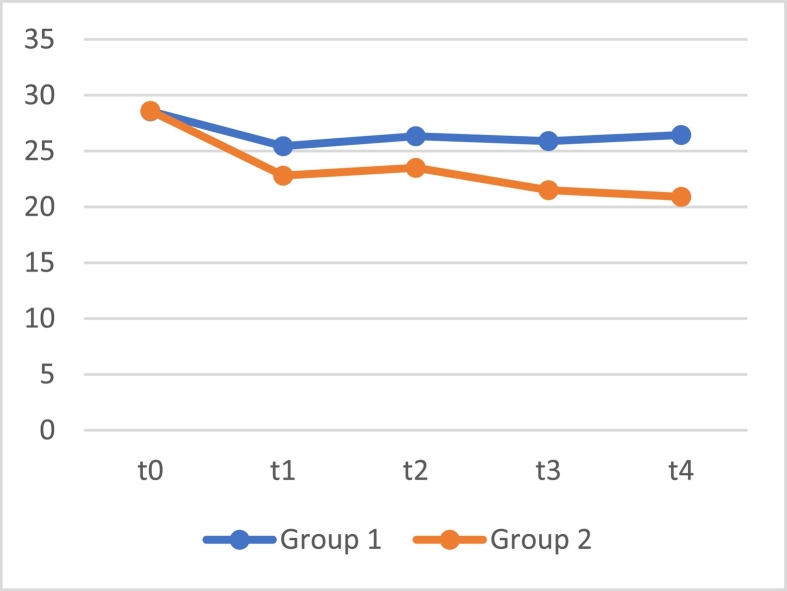
The BAQ scores of Group 1 (app, e-mail, app) and Group 2 (e-mail, app, e-mail) over time, with a significant main effect for time (*F*[4, 68.000] = 3.311, *p* = .015).

**Table A3:** Outcomes of the inductive analysis of interviews about the experiences of 10 randomly selected participants that completed the study.

Code	Explanation	Example quote	Participants (N = 10)
Perceptions of the intervention	Participants noted changes in their cognitions because they had to be more conscious about using their non-dominant hand	“*I had to use other functions of my brain that I felt like they were asleep*. *I had to pay more attention to the things I did in my daily life* (*pp*. *6*)”.	6
Comparison between the app and e-mail instructions	Most participants preferred the app over the e-mail instructions (*n* = 8), mainly because the app sent them reminders to support the use of the non-dominant hand	“*I prefer the app because I use my phone every day* (…) *the app is more convenient* (*pp*. *9*)”.	8
Bugs in the app	Some participants experienced different types of bugs while using the app, ranging from unusable (not logging in) to slightly annoying (app automatically closed once).	“*Only once it bugged* (…), *I could not type*. *But otherwise*, *everything was normal*. *I really enjoyed it* (*pp*. *2*)”.	4
Points of improvement	While participants generally liked the app, they identified some points of improvements: more reminders for tasks; more visually attractive; need for more personalization in terms of type and number of tasks; following progress	“*I'd definitely make sure it tracks progress* (…) *so you'd see which tasks you've done and if you're doing great or not* (*pp*. *4*)”.	6

**Table B1:** The codes of the opinions of the participants and number of times they were mentioned by all participants that received the app (n = 68) and e-mail (n = 68), and received either one or five tasks within that condition.

Codes	Total	1 task (*n* = 34)	5 tasks (n = 34)
App (n = 68)			
Positive			
App was easy to use and clear	26	12	14
Design of the app was appealing	13	4	9
Positive evaluation, without further explanation	11	9	2
The app was a good reminder of the tasks	10	5	5
The app functioned as it should/no bugs	9	6	3
App was fun to use	4	1	3
Nice that the app mentions your name	1	1	0
Negative			
Bugs in answering questions	34	18	16
Not enough reminders were sent	9	3	6
Small elements of the app were unclear	5	2	3
Not able to easily look up tasks	4	2	2
Design can be improved	2	1	1
Receiving five tasks at once was too much	1	0	1

E-mail (n = 68)			
Positive			
The content of the e-mails was easy to understand	40	18	22
The overview of timeline was clear	11	3	8
The design of the e-mails was appealing	8	6	2
Positive evaluation, without further explanation	8	6	2
E-mails served as a reminder of the tasks	6	2	4
The tone of the e-mails was friendly	4	0	4
The e-mails were sent at a convenient time (8 a.m.)	4	3	1
Opportunity to ask questions to the researcher	2	0	2
E-mails contained the right amount of information	1	0	1
Negative			
Not enough reminders were sent	8	5	3
Small elements of the e-mails were unclear	4	1	3
Design of the e-mails can be improved	3	2	1
The e-mails were sent at an inconvenient time (8 a.m.)	3	3	0
Text of the e-mails was not visible on phone	2	1	1

**Table C1:** The points of improvement and number of times they were mentioned by all participants that received SCT (N = 134) and for the e-mail and app group, and the 1 and 5 task group (each n = 68).

Code	Total	App (% of total)	E-mail (% of total)	5 tasks (% of total)	1 task (% of total)
No points of improvement	40	19 (47.5%)	21 (52.5%)	15 (37.5%)	25 (62.5%)
Add more reminders to perform the tasks	32	14 (43.8%)	18 (56.2%)	20 (62.5%)	12 (37.5%)
Change one or more of the tasks	19	13 (68.4%)	6 (31.6%)	14 (73.7%)	5 (26.3%)
Fix the bugs in the app	7	7 (100%)	0 (0%)	3 (42.9%)	4 (57.1%)
Add a validation/way to prevent ‘cheating’	6	2 (33.3%)	4 (66.7%)	1 (16.7%)	5 (83.3%)
Provide only one task per day	5	3 (60%)	2 (40%)	5 (100%)	0 (0%)
Provide more than one task per day	4	2 (50%)	2 (50%)	0 (0%)	4 (100%)
More explanation on the goal of the intervention	4	2 (50%)	2 (50%)	1 (25%)	3 (75%)
Make it easier to see the tasks in the app	2	1 (50%)	1 (50%)	1 (50%)	1 (50%)
Change the time when the e-mail was sent	2	0 (0%)	2 (100%)	0 (0%)	2 (100%)
Suggestions for changes to the study design	24	10 (41.7%)	14 (58.3%)	12 (50%)	12 (50%)

**Table D1:** Participants' assessment of how well they performed on the tasks, how difficult the tasks were on a Likert scale^a^, and the codes for the reasons for removal.

Task #	Content	How well? Mode; Median (n = 68)	Difficulty? Mode; Median (*n* = 68)	# remove (*N* = 135)	Reason for removal
10	Write	1; 1	5; 5	84 (62.22%)	Inconvenient for daily life (*n* = 54); Too difficult/hard to maintain (*n* = 26); Task not very common (n = 4).
3	Use cards	3; 3	4; 3	33 (24.44%)	Task not very common (*n* = 12); Too difficult/hard to maintain (n = 7); Inconvenient for daily life (*n* = 7); Using non-dominant hand or both hands anyway (*n* = 3); Too easy (*n* = 2); Task description unclear (*n* = 1).
8	Use mobile phone	2; 3	4; 4	32 (23.70%)	Too difficult/hard to maintain (n = 20); Task not very common (*n* = 7); Using non-dominant hand or both hands anyway (*n* = 5).
1	Switch lights on/off	4; 4	1; 2	22 (16.30%)	Too easy (*n* = 11); Task description unclear (*n* = 6); Using non-dominant hand or both hands anyway (*n* = 5); Too difficult/hard to maintain (*n* = 3); Task not very common (n = 2).
7	Pick-up and carry items	3; 3	2; 2	22 (16.30%)	Task not very common (n = 7); Too difficult/hard to maintain (*n* = 6); Using non-dominant hand or both hands anyway (*n* = 6); Task description unclear (n = 3).
9	Eat	3; 3	4; 4	19 (14.07%)	Too difficult/hard to maintain (*n* = 11); Inconvenient for daily life (n = 5); Using non-dominant hand or both hands anyway (n = 3).
2	Open/close zippers and buttons	3; 3	4; 4	17 (12.59%)	Using non-dominant hand or both hands anyway (n = 9); Too difficult/hard to maintain (*n* = 4); too easy (*n* = 2); Task not very common (*n* = 2); Task description unclear (*n* = 1).
4	Press buttons	4; 4	2; 2	15 (11.11%)	Too easy (n = 6); Task description unclear (*n* = 5); Task not very common (n = 2); Using non-dominant hand or both hands anyway (n = 2).
6	Open doors	4; 4	2; 2	15 (11.11%)	Using non-dominant hand or both hands anyway (*n* = 9); Too easy (*n* = 4); Too difficult/hard to maintain (n = 2).
5	Drink using a cup or a mug, bottle	4; 4	2; 2	2 (1.48%)	Inconvenient for daily life (n = 1); Using non-dominant hand or both hands anyway (n = 1).

a 1 = Not well/difficult at all; 2 = not very well/difficult; 3 = moderately well/difficult; 4 = very well/difficult; 5 = extremely well/difficult.

## References

[bb0005] Andersson G., Estling F., Jakobsson E., Cuijpers P., Carlbring P. (2011). Can the patient decide which modules to endorse? An open trial of tailored internet treatment of anxiety disorders. Cogn. Behav. Ther..

[bb0010] Baumeister R.F., Vohs K.D., Tice D.M. (2007). The strength model of self-control. Curr. Dir. Psychol. Sci..

[bb0015] Beames J., Schofield T.P., Denson T.F., de Ridder D.T., Adriaanse M., Fujita K. (2017). A meta-analysis of improving self-control with practice. (red.) International Handbook of Self-Control in Health and Well-Being.

[bb0020] Berkman E.T. (2016). Self-regulation training. Handbook of Self-Regulation: Research, Theory, and Applications.

[bb0025] Boeije H. (2002). A purposeful approach to the constant comparative method in the analysis of qualitative interviews. Qual. Quant..

[bb0030] Brouwer W., Kroeze W., Crutzen R., De Nooijer J., de Vries N.K., Brug J., Oenema A. (2011). Which intervention characteristics are related to more exposure to internet-delivered healthy lifestyle promotion interventions? A systematic review. J. Med. Internet Res..

[bb0035] Brown M., O'neill N., Van Woerden H., Eslambolchilar P., Jones M., John A. (2016). Gamification and adherence to web-based mental health interventions: a systematic review. JMIR Ment. Health.

[bb0040] Burns C. (2018). Human-centred design. eHealth Research, Theory and Development.

[bb0045] Clausen W., Watanabe-Galloway S., Baerentzen M.B., Britigan D.H. (2016). Health literacy among people with serious mental illness. Community Ment. Health J..

[bb0050] Collins L.M., Kugler K.C. (2018). Optimization of Behavioral, Biobehavioral, and Biomedical Interventions.

[bb0055] Collins L.M., Dziak J.J., Kugler K.C., Trail J.B. (2014). Factorial experiments: efficient tools for evaluation of intervention components. Am. J. Prev. Med..

[bb0060] Da Silva M.C. (2019). A Mobile App-Based Intervention for Self-Control (Hands-ON): Usability and Feasibility Evaluations.

[bb0065] Dallery J., Cassidy R.N., Raiff B.R. (2013). Single-case experimental designs to evaluate novel technology-based health interventions. J. Med. Internet Res..

[bb0070] De Ridder D.T., Lensvelt-Mulders G., Finkenauer C., Stok F.M., Baumeister R.F. (2012). Taking stock of self-control: a meta-analysis of how trait self-control relates to a wide range of behaviors. Personal. Soc. Psychol. Rev..

[bb0075] Deenik J., Tenback D.E., Tak E.C., Henkemans O.A.B., Rosenbaum S., Hendriksen I.J., Van Harten P.N. (2019). Implementation barriers and facilitators of an integrated multidisciplinary lifestyle enhancing treatment for inpatients with severe mental illness: the MULTI study IV. BMC Health Serv. Res..

[bb0080] Del Vecchio T., O’Leary K.D. (2004). Effectiveness of anger treatments for specific anger problems: a meta-analytic review. Clin. Psychol. Rev..

[bb0085] Denson T.F., Capper M.M., Oaten M., Friese M., Schofield T.P. (2011). Self-control training decreases aggression in response to provocation in aggressive individuals. J. Res. Pers..

[bb0090] Denson T.F., Dewall C.N., Finkel E.J. (2012). Self-control and aggression. Curr. Dir. Psychol. Sci..

[bb0095] Dijk J.V., Kesteren J.V., Smit P. (2007). Criminal Victimisation in International Perspective, Boom Juridische Uitgevers.

[bb0100] Dodge K.A., Coie J.D. (1987). Social-information-processing factors in reactive and proactive aggression in children’s peer groups. J. Pers. Soc. Psychol..

[bb0105] Drieschner K.H., Boomsma A. (2008). The treatment motivation scales for forensic outpatient treatment (tms-f) construction and psychometric evaluation. Assessment.

[bb0110] Duckworth A.L., Kern M.L. (2011). A meta-analysis of the convergent validity of self-control measures. J. Res. Pers..

[bb0115] Finkel E.J., Dewall C.N., Slotter E.B., Oaten M., Foshee V.A. (2009). Self-regulatory failure and intimate partner violence perpetration. J. Pers. Soc. Psychol..

[bb0120] Friese M., Frankenbach J., Job V., Loschelder D.D. (2017). Does self-control training improve self-control? A meta-analysis. Perspect. Psychol. Sci..

[bb0125] Fung S.-F., Kong C.Y.W., Huang Q. (2019). Evaluating the dimensionality and psychometric properties of the brief self-control scale amongst Chinese university students. Front. Psychol..

[bb0130] Gaynes B.N., Brown C.L., Lux L.J., Brownley K.A., Van Dorn R.A., Edlund M.J., Coker-Schwimmer E., Weber R.P., Sheitman B., Zarzar T. (2017). Preventing and de-escalating aggressive behavior among adult psychiatric patients: a systematic review of the evidence. Psychiatr. Serv..

[bb0135] Geoffrion S., Goncalves J., Sader J., Boyer R., Marchand A., Guay S. (2017). Workplace aggression against health care workers, law enforcement officials, and bus drivers: differences in prevalence, perceptions, and psychological consequences. J. Work. Behav. Health.

[bb0140] Gottfredson M.R., Hirschi T. (1990). A General Theory of Crime.

[bb0145] Greenberg E., Dunleavy E., Kutner M. (2007). Literacy Behind Bars: Results from the 2003 National Assessment of Adult Literacy Prison Survey. NCES 2007-473.

[bb0150] Hagger M.S., Wood C., Stiff C., Chatzisarantis N.L. (2010). Ego depletion and the strength model of self-control: a meta-analysis. Psychol. Bull..

[bb0155] Kelders S.M., Kok R.N., Ossebaard H.C., Van Gemert-Pijnen J.E. (2012). Persuasive system design does matter: a systematic review of adherence to web-based interventions. J. Med. Internet Res..

[bb0160] Kobes M.H., Nijman H.H., Bulten E.B. (2012). Assessing aggressive behavior in forensic psychiatric patients: validity and clinical utility of combining two instruments. Arch. Psychiatr. Nurs..

[bb0165] Krasny-Pacini A., Evans J. (2018). Single-case experimental designs to assess intervention effectiveness in rehabilitation: a practical guide. Ann. Phys. Rehab. Med..

[bb0170] Krug E.G., Mercy J.A., Dahlberg L.L., Zwi A.B. (2002). The world report on violence and health. Lancet.

[bb0175] Lindner C., Nagy G., Retelsdorf J. (2015). The dimensionality of the brief self-control scale—an evaluation of unidimensional and multidimensional applications. Personal. Individ. Differ..

[bb0180] Ludden G.D., Van Rompay T.J., Kelders S.M., Van Gemert-Pijnen J.E. (2015). How to increase reach and adherence of web-based interventions: a design research viewpoint. J. Med. Internet Res..

[bb0185] Michie S., Yardley L., West R., Patrick K., Greaves F. (2017). Developing and evaluating digital interventions to promote behavior change in health and health care: recommendations resulting from an international workshop. J. Med. Internet Res..

[bb0190] Mohr D.C., Schueller S.M., Riley W.T., Brown C.H., Cuijpers P., Duan N., Kwasny M.J., Stiles-Shields C., Cheung K. (2015). Trials of intervention principles: evaluation methods for evolving behavioral intervention technologies. J. Med. Internet Res..

[bb0195] Muraven M., Baumeister R.F., Tice D.M. (1999). Longitudinal improvement of self-regulation through practice: building self-control strength through repeated exercise. J. Soc. Psychol..

[bb0200] Oinas-Kukkonen H., Harjumaa M. (2009). Persuasive systems design: key issues, process model, and system features. Commun. Assoc. Inf. Syst..

[bb0205] Pekurinen V., Willman L., Virtanen M., Kivimäki M., Vahtera J., Välimäki M. (2017). Patient aggression and the wellbeing of nurses: a cross-sectional survey study in psychiatric and non-psychiatric settings. Int. J. Environ. Res. Public Health.

[bb0210] Pfeffer I., Strobach T. (2017). Executive functions, trait self-control, and the intention–behavior gap in physical activity behavior. J. Sport Exerc. Psychol..

[bb0215] Polaschek D.L., Wilson N.J., Townsend M.R., Daly L.R. (2005). Cognitive-behavioral rehabilitation for high-risk violent offenders: an outcome evaluation of the violence prevention unit. J. Interpersonal Violence.

[bb0220] Poulin F., Boivin M. (2000). Reactive and proactive aggression: evidence of a two-factor model. Psychol. Assess..

[bb0225] Ross J., Quayle E., Newman E., Tansey L. (2013). The impact of psychological therapies on violent behaviour in clinical and forensic settings: a systematic review. Aggress. Violent Behav..

[bb0230] Rutherford A., Zwi A.B., Grove N.J., Butchart A. (2007). Violence: a priority for public health?(part 2). J. Epidemiol. Community Health.

[bb0235] Sailer M., Hense J., Mandl J., Klevers M. (2014). Psychological perspectives on motivation through gamification. Interact. Des. Archit. J..

[bb0240] Saini M. (2009). A meta-analysis of the psychological treatment of anger: developing guidelines for evidence-based practice. J. Am. Acad. Psychiatry Law Online.

[bb0245] Sniehotta F.F., Scholz U., Schwarzer R. (2005). Bridging the intention–behaviour gap: planning, self-efficacy, and action control in the adoption and maintenance of physical exercise. Psychol. Health.

[bb0250] Sniehotta F., Presseau J., Araújo-Soares V. (2014). Time to retire the theory of planned behaviour. Health Psychol. Rev..

[bb0255] Tangney J.P., Baumeister R.F., Boone A.L. (2004). High self-control predicts good adjustment, less pathology, better grades, and interpersonal success. J. Pers..

[bb0260] Van Dijk J., Manchin R., Van Kesteren J., Hideg G. (2007). The burden of crime in the EU. A Comparative Analysis of the European Survey of Crime and Safety (EU ICS 2005).

[bb0265] Van Gemert-Pijnen J.E., Nijland N., Van Limburg M., Ossebaard H.C., Kelders S.M., Eysenbach G., Seydel E.R. (2011). A holistic framework to improve the uptake and impact of eHealth technologies. J. Med. Internet Res..

[bb0270] Van Gemert-Pijnen L.J., Kip H., Kelders S.M., Sanderman R. (2018). Introducing ehealth. eHealth Research, Theory and Development.

[bb0275] Webster G.D., Dewall C.N., Pond R.S., Deckman T., Jonason P.K., Le B.M., Nichols A.L., Schember T.O., Crysel L.C., Crosier B.S. (2015). The brief aggression questionnaire: structure, validity, reliability, and generalizability. J. Pers. Assess..

[bb0280] Westermann T., Möller S., Wechsung I. (2015). Assessing the relationship between technical affinity, stress and notifications on smartphones. Proceedings of the 17th International Conference on Human-Computer Interaction with Mobile Devices and Services Adjunct.

